# An ion-imprinted thiocyanato-functionalized mesoporous silica for preconcentration of gold(III) prior to its quantitation by slurry sampling graphite furnace AAS

**DOI:** 10.1007/s00604-018-3106-x

**Published:** 2018-11-28

**Authors:** Joanna Dobrzyńska, Marzena Dąbrowska, Rafał Olchowski, Ryszard Dobrowolski

**Affiliations:** Department of Analytical Chemistry and Instrumental Analysis, Faculty of Chemistry, Maria Curie-Sklodowska University, M. C. Sklodowska Sq. 3, 20-031 Lublin, Poland

**Keywords:** Gold adsorption; ion-imprinted silica; solid phase extraction; functionalized SBA-15; graphite furnace atomic absorption spectrometry, One-pot synthesis, Sol-gel, Gold determination, Ordered mesoporous silicas, Gold imprinting

## Abstract

A gold(III)-imprinted thiocyanato-functionalized silica network of type SBA-15 was prepared by co-condensation of tetraethoxysilane (TEOS) with thiocyanatopropyltriethoxysilane (TCTES) in the presence of Pluronic123 and Au(III) ions. Compared to the non-imprinted material, the imprint has a higher selectivity and adsorption capacity for Au(III). The maximum static adsorption capacity for Au(III) is 475 mg·g^−1^ for the ion-imprinted, and 62 mg·g^−1^ for the non-imprinted sorbent. The imprint was applied to the sorption of Au(III) from digested geological samples prior to its determination by graphite furnace atomic absorption spectrometry. Adsorption is fast and does not substantially prolong the analytical procedure. Under optimum conditions, the detection limit for Au(III) is 2 ng·g^−1^. The method was validated by analyzing certified reference materials, and results were in good agreement with certified values. The procedure was successfully applied to the separation and determination of gold in complex geological samples.

**Electronic supplementary material:**

The online version of this article (10.1007/s00604-018-3106-x) contains supplementary material, which is available to authorized users.

**Graphical abstract:**
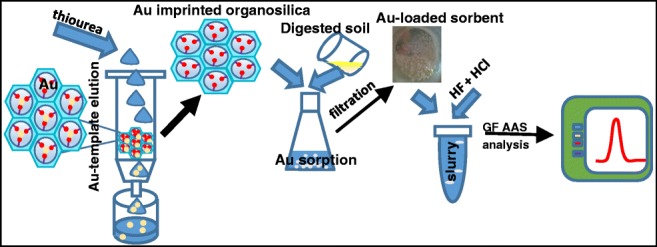
Schematic presentation of the preparation of ion-imprinted thiocyanato-functionalized mesoporous silica and its application for the preconcentration of gold from digested soils before its determination by slurry sampling graphite furnace atomic absorption spectrometry (GF AAS).

## Introduction

The numerous applications of gold, which is classified as a toxic element, necessitate the development of reliable and precise analytical procedures for its determination in various environmental samples [1].

Trace level determinations of gold are usually performed by using atomic absorption spectrometry (AAS) [2], inductively coupled plasma atomic emission spectrometry (ICP-OES) [3] and inductively coupled plasma mass spectrometry (ICP-MS) [4]. However, in many cases the presence of a complex sample matrix precludes obtaining reliable results by direct application of those spectrometric techniques. Extensive and difficult to eliminate interferences often necessitate separation/preconcentration of the analyte prior to its determination [5]. Among the separation techniques [6–11] used for preconcentration of gold solid phase extraction (SPE) seems to be especially attractive [12]. Various sorbents like nanotubes [13], activated carbon [14], Amberlite XAD resins [15], polyurethane foams [16], nanoclays [17] and silica gels [18] have been used for enrichment of Au(III) ions from different media.

Due to high selectivity towards the target ions, ion-imprinted polymers have gained popularity [19]. Synthesis of these materials is based on interactions between the imprinted ions and the functional monomers. Functional monomers agglomerate around the template creating a three-dimensional complex, which is joined with the polymeric matrix during the polymerization process. The cross-linking monomer or other matrix-making material is responsible for the spatial locking of the produced complex and the rigidity of the polymer, which determines the stability of the recognition sites. Subsequently the material is subjected to intense washing steps in order to remove the template. The selectivity of the polymeric material is related to the charge, coordination geometry, coordination number and size of the target ion [20]. Ion-imprinted polymers have various desireable properties such as: the high recognition, chemical and mechanical stability, ease and low cost of preparation as well as high adsorption selectivity towards specific analytes. However, these materials are characterized by low active surface, which confers low adsorption capacities towards specific metal ions [21].

To overcome this limitation the mesoporous organosilica matrix has been applied in this study to make new ion-imprinted materials. This concept is based upon ordered mesoporous silicas (like SBA-15), which have the desirable properties: high surface area (up to 1000 m^2^ g^−1^), large pore sizes and volumes (up to 30 nm, 1.3 cm^3^ g^−1^, respectively), good mechanical and chemical stability related to the thick pore walls (3.1–6.4 nm) and the homogeneity of pores [22]. Also the surface of the mesoporous silica can be easily modified. Since silica is a non-swelling material, unlike organic polymers, the recognition sites present on its surface are more stable, which results in higher selectivity towards target ions [23]. Ion-imprinted mesoporous organosilicas can be prepared using a sol-gel or grafting method. In the first case functional monomers co-condense with a silica precursor in the presence of imprinted ions, while in the latter mesoporous silica obtained in the presence of an ionic template is subjected to functionalization. The synthesis performed in the presence of the ionic template leads to the creation of recognition sites in the framework of the mesoporous silica sorbents [24].

The current study investigated the adsorption of gold ions on the Au(III)-imprinted thiocyanato-functionalized SBA-15 followed by slurry sampling GF AAS determination. The impact of basic parameters on the adsorption capacity of gold ions onto Au(III)-imprinted modified SBA-15 such as contact time, pH of the sample and presence of chosen co-existing ions was investigated. The optimized conditions were then applied for Au(III) enrichment from solutions of digested geological samples before Au determination by slurry sampling GF AAS. Certified reference materials were used to verify and validate the procedure.

## Experimental

### Reagents and materials

The following compounds were used: tetraethoxysilane (TEOS, 99%, ABCR, Karlsruhe, Germany, www.abcr.de), thiocyanatopropyltriethoxysilane (TCTES, 95%, Sigma-Aldrich, Poznan, Poland, www.sigmaaldrich.com), Pluronic P123 (P123, Sigma-Aldrich, Poznan, Poland, www.sigmaaldrich.com), hydrochloric acid (Merck, Darmstadt, Germany, www.merckgroup.com), hydrofluoric acid, nitric acid (Merck, Darmstadt, Germany, www.merckgroup.com), standard solutions of Pt(IV) (1000 mg L^−1^, Merck, Darmstadt, Germany, www.merckgroup.com), Au(III) (1000 mg L^−1^, Merck, Darmstadt, Germany, www.merckgroup.com), Pd(II) (1000 mg L^−1^, Merck, Darmstadt, Germany, www.merckgroup.com) and Ru(III) (1000 mg L^−1^, Merck, Darmstadt, Germany, www.merckgroup.com), gold chloride (99%, Sigma-Aldrich, Poznan, Poland, www.sigmaaldrich.com), ethanol (EtOH, 99.8%, POCH, Gliwice, Poland, http://www.poch.com.pl), sodium hydroxide (Merck, Darmstadt, Germany, www.merckgroup.com), sodium chloride (Acros Organics, Geel, Belgium, www.acros.com), potassium nitrate (POCH, Gliwice, Poland, http://www.poch.com.pl), thiourea (99%, Sigma-Aldrich, Poznan, Poland, www.sigmaaldrich.com). Throughout all analytical work, Milli-Q water was used (Millipore, Darmstadt, Germany, http://www.merckmillipore.com).

The certified reference materials Ma–2b (CANMET, Ottawa, Ontario, www.nrcan.gc.ca), SARM–7 (IAEA, Vienna, www.iaea.org), SRM 886 (NIST, Gaithersburg, Maryland, www.nist.gov) and WPR–1 (CANMET, Ottawa, Ontario, www.nrcan.gc.ca) were used to verify and validate the procedure. Samples of copper shale (POLK I and POLK II) and magmatic rocks (PIG I, PIG II, PIG III and PIG IV) were obtained from the Central Laboratory of the Polish Geological Institute (www.pgi.gov.pl).

### Instruments

Nitrogen adsorption/desorption isotherms were measured at −196 °C using an ASAP-2405 N analyzer (Micromeritics Corp., Norcross, Georgia, USA, www.micromeritics.com). Powder X-ray diffraction (XRD) patterns were recorded using a Empyrean (Panalytical, Almelo, Netherlands, www.malvernpanalytical.com) diffractometer (CuKα radiation) with 0.02° size step and 10 s time step covering the range of 0.5 < 5.0° at room temperature. X-ray photoelectron spectroscopy (XPS) spectra were collected using a Multi-Chamber Analytical System (Prevac, Rogów, Poland, www.prevac.pl) with monochromated Kα Al radiation (1486.6 eV) (Gammadata, Scienta, Uppsala, Sweden, www.scienta.se) and an X-ray power of 450 W. The binding energy scale was referenced against C1s = 284.7 eV line. The vacuum in the analysis chamber was better than 1.5 ∙ 10^−7^ Pa.

Measurements of gold concentrations in the liquid phase of the studied adsorption systems, depending on the gold concentration, were carried out using a Varian (Mulgrave, Victoria, Australia, www.agilent.com) SpectrAA atomic absorption spectrometer equipped with air/acetylene flame or Varian (Mulgrave, Victoria, Australia, www.agilent.com) SpectrAA 800 atomic absorption spectrometer equipped with a GTA 100 graphite furnace and Zeeman background correction. The temperature program used for determination of gold in liquids and slurries was: drying: 120 °C for 35 s, pyrolysis: 1000 °C for 10 s, atomization: 2600 °C for 5 s.

### Synthesis of adsorbent

Ion-imprinted materials were synthesized via a sol-gel process by a one-pot route synthesis. In the model synthesis, 2 g of P123 was dissolved in 72 mL of 1.6 mol L^−1^ HCl under vigorous stirring at 40 °C. After 8 h of stirring 0.15 g of gold chloride was added. After dissolution of the solid 18 mmol (19 mmol) of TEOS was added dropwise, followed by 2 mmol (1 mmol) of TCTES. The resulting mixture was stirred for 24 h at 40 °C and aged at 100 °C for next 48 h. The solids were filtered. Finally, the solid material was purified from Pluronic123 by triple extraction with acidified ethanol (99.8%) at 78 °C, whereas the template Au(III) ions were removed from the material using 0.5 mol L^−1^ thiourea in 5% HCl, until Au was not detected in the leaching solution. The process was monitored by GF AAS. The control sorbents were similarly synthesized but in absence of gold chloride. The ion-imprinted materials were labeled **Au(III)/S1**(TEOS:TCTES 18:2) and **Au(III)/S2** (TEOS:TCTES 19:1), whereas non-imprinted materials were labeled **S1** (TEOS:TCTES 18:2) and **S2** (TEOS:TCTES 19:1).

### Adsorption and selectivity measurement

In every adsorption experiment, 50 mg of adsorbent and 50 mL of Au(III) solution were stirred at 25 ± 0.5 °C. After the adsorption equilibrium had been attained, the solution was separated from the sorbent by centrifugation and gold was determined by F AAS.

The adsorption of Au(III) onto studied materials *a* (mg g^−1^), was calculated as follows:

1 $$ a=\frac{\left({c}_i-c\right)\bullet V}{m} $$

where *c*_*i*_ is the initial Au(III) concentration (mg L^−1^), *c* is the equilibrium Au(III) concentration (mg L^−1^), *V* is the volume of the solution (L) and *m* is the mass of the adsorbent (g).

The selectivity of Au(III) adsorption in the presence of Pd(II), Pt(IV) and Ru(III) ions was tested for **Au(III)/S1** and **S1** sorbents using the same concentrations of Au(III) and competitive ions. The distribution coefficients of Pd(II), Pt(IV), Ru(III) and Au(III) ions can be determined as follows:

2 $$ {K}_d=\left[\left({c}_i-c\right)/c\right]\left(V/m\right) $$

where *c*_*i*_ is the initial concentration (mg L^−1^), *c* is the equilibrium concentration (mg L^−1^), *V* is the volume of the solution (L) and *m* is the mass of the adsorbent (g).

The selectivity coefficient for the sorption Au(III) in the presence of competition ions is given as:

3 $$ k={K}_d\left( Au(III)\right)/{K}_d(X) $$

where X represents Pd(II), Ru(III), Pt(IV). Selectivity coefficient represents Au(III) adsorption selectivity when there are other metal ions present in aqueous solution. A larger *k* suggest a higher selectivity for the Au(III) ions. A relative selectivity coefficient can be defined as:

4 $$ {k}^{\prime }={k}_{imprinted}/{k}_{non- imprinted} $$

A higher relative selectivity coefficient indicates stronger adsorption affinity and better selectivity of imprinted adsorbents for the Au(III) ions compared to non-imprinted material.

### Determination of gold

For the determination of gold in geological samples (POLK I, POLK II, PIG I, PIG II, PIG III, PIG IV) and certified reference materials, 0.1 g of a given dried sample was weighed, placed into Teflon vessel and treated with 10 mL of *aqua regia*. Samples were digested in the microwave system (Mars 5, CEM Corp, Matthews, North Carolina, USA, http://cem.com) at 190 °C and 1.24 MPa. The digested samples were made up to 50 mL with Milli-Q water and transferred to 100 mL beakers and evaporated almost to dryness. Subsequently 2 mL of 30% HCl were added and samples were again evaporated, with this step repeated several times to convert gold to chloride complexes. The blanks were prepared in the same way. The samples were then made up to 50 mL with Milli-Q water and pH was adjusted to 2 by addition of sodium hydroxide. The solutions were shaken with 0.02 g of the **Au(III)/S1** for 3 h. The Au-loaded sorbent was separated from the solution by filtration and dried at 105 °C to constant weight. The slurries for GF AAS analysis were prepared by mixing of 0.01 g of dried Au-loaded **Au(III)/S1** with 0.1 mL of 40% HF in Eppendorf vessels and after 15 min with 0.9 mL of 5% HCl. Since the complete dissolution of sorbent was achieved the contents of Eppendorf vessels were not homogenized before GF AAS analysis.

## Results

### Choice of materials

The synthesis of Au(III) ion-imprinted thiocyanato-functionalized silicas of SBA-15 type was undertaken because the designed material was expected to have high specific surface area, high hydrothermal stability, easily accessible adsorption sites and exhibit high selectivity and adsorption capacity for Au(III) ions. The advantage of the Au(III) ion-imprinted thiocyanato-functionalized silicas synthesized in the presence of P123 micelles are the wide, cylindrical, hexagonally ordered mesopores ensuring the effective diffusion of the adsorbate and homogenously distributed Au(III) shape-matched adsorption centers. Due to the presence of the silica skeleton, the materials do not swell, which means that the adsorption centers are more stable than in the case of polymer sorbents. The important aspect influencing the synthesis of ion-imprinted SBA-15 is the simplicity of the preparation described in the “Synthesis of adsorbent” section. In the reaction mixture Pluronic 123 triblock copolymer forms a hexagonal micellar template on which surface co-condensation of TEOS and TCTES in the presence if AuCl_3_ occurs. SBA-15 material with pores filled with P123 and –SCN groups bonded with gold is obtained. To remove P123 and gold templates, acidified ethanol and thiourea are used, respectively.

### Characterization

The porosity and the degree of order of the materials were monitored by measurements of nitrogen adsorption/ desorption isotherms and XRD analysis (See Electronic Supporting Material (ESM)).

As presented in Table 1, the values of S_BET_ for S1 and S2 are equal to 683 and 668 m^2^ g^−1^, respectively. Although the increase of the amount of TCTES in the reaction mixture used for synthesis does not cause a decrease of the active surface, it leads to the diminution of the pore volumes and their diameter.

**Table 1:** Structure-adsorption characteristics of the synthesized materials

Material	Molar composition	S_BET_^a^ [m^2^ g^−1^]	V_p_^b^ [cm^3^ g^−1^]	d_BJH_^c^ [nm]
S1	TEOS:TCTES	683	0.66	4.5
	18:2			
Au(III)/S1	TEOS:TCTES	290	0.20	3.7
	18:2			
S2	TEOS:TCTES	668	0.77	5.4
	19:1			
Au(III)/S2	TEOS:TCTES	389	0.34	4.1
	19:1			

^a^Specific surface area determined by BET method

^b^Total pore volume for the relative pressure of 0,99

^c^Average pore size calculated from adsorption branch of isotherm by BJH method

The addition of AuCl_3_ to the reaction mixture results in a collapsed porous structure. However, the S_BET_ of **Au(III)/S1** and **Au(III)/S2** is still high and equal to 290 and 389 m^2^ g^−1^, respectively.

Based on the XRD patterns (See ESM) for pristine SBA-15 and thiocyanato-functionalized materials **S1** and **S2** it can be concluded that the increase of the molar ratio of TCTES results in deterioration of the ordering of the material. Moreover, the addition of Au(III) ions leads to further deterioration of the degree of order, that is reflected in the absence of the peak chcaracteristic for hexagonall *P6mm* symmetry on the **Au(III)/S1** pattern.

### Adsorption conditions optimisation

In order to ensure the highest adsorption of Au(III) ions from digested geological samples onto synthesized materials the pH value of the solution and contact time were optimized (ESM). In short, the following experimental conditions were found to give the best adsorption results:

- pH value
  The adsorption of Au(III) onto **Au(III)/S1** and **Au(III)/S2** is very high in the pH range 0.5 to 4.2. In the case of **S1** adsorption is also high in the pH range between 2.0 and 4.2, whereas for **S2** highest adsorption values are obtained in the pH range between 3.0 and 4.2. For further adsorption experiments with application of **S1**, **Au(III)/S1** and **Au(III)/S2** the selected pH was 2.0 and for **S2** pH selected was 3.0.
- contact time
  For sorbents **S2** and **Au(III)/S2** 10 h are required to reach adsorption equilibrium. In the case of **Au(III)/S1** and **S1** the times needed for reaching equilibrium are about 3 and 20 h, respectively.

### Adsorption capacity

**Fig. 1:**
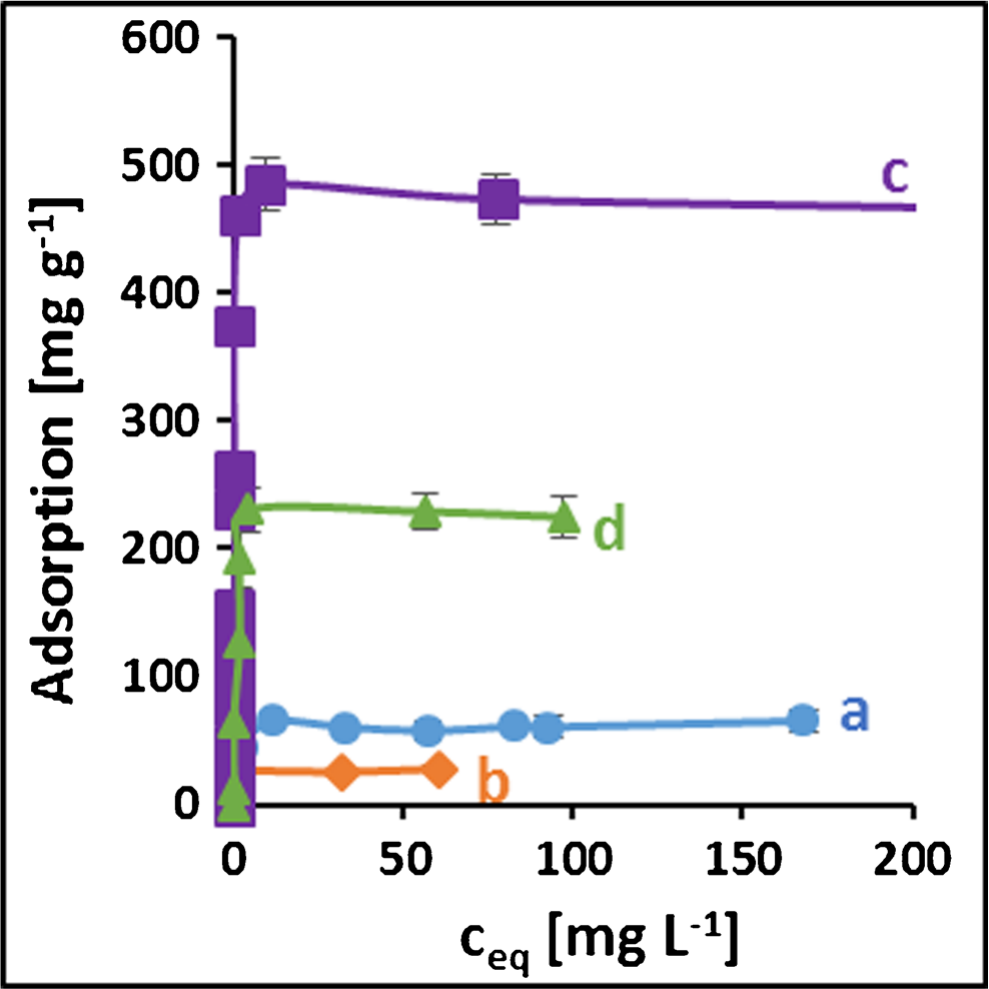
Adsorption isotherms of Au(III) onto **a** – S1, **b** - S2, **c –** Au(III)/S1, **d –** Au(III)/S2 (m = 50 mg, V = 50 mL, t_S1_ = 20 h, t_Au(III)/S1_ = 3 h, t_S2, Au(III)S2_ = 10 h, pH_S1,Au(III)S1,Au(III)S2_ = 2, pH_S2_ = 3, T = 25 °C)

The adsorptions isotherms of Au(III) from aqueous solutions onto studied thiocyanato-functionalized materials are shown in Fig. 1. The adsorption capacities are directly proportional to the amount of TCTES used for sorbent synthesis. Thus the number of the adsorption sites is most likely correlated with the concentration of -SCN groups on the sorbent surface. However, their accessibility for Au(III) ions is probably limited and for this reason the key factor affecting the sorption ability and capacity of sorbent is the presence of Au(III) ion-imprinted sites, which eliminate the steric hindrance and allow for efficient binding of Au(III) with the sorbent surface. The maximum static adsorption capacity of the imprinted materials is above 7 times higher than that for non-imprinted sorbents. The highest adsorption of 475 mg g^−1^ was obtained for **Au(III)/S1** sorbent; this value significantly exceeds adsorption capacities reported in the literature and summarized in Table 3. Based on its highest adsorption capacity, **Au(III)/S1** material was chosen for subsequent analytical applications.

### Effect of interfering ions

Due to the common use of *aqua regia* for digestion of analysed solid samples the influence of nitrates and chlorides on the adsorption of Au(III) was also investigated for **Au(III)/S1** and **S1** materials. It was found that the presence of chlorides and nitrates does not hinder Au(III) adsorption from solutions obtained after acidic digestion of environmental solid samples. Detailed information concenrning the effect of the above-mentioned anions on the Au(III) adsorption is presented in the ESM.

### Desorption studies

The desorption of Au using different concentrations of hydrochloric acid, nitric acid and thiourea was performed by applying the batch method. The Au desorption efficiency from **Au(III)/S1** material was 18% for 10 mol L^−1^ HCl, 33% for 14 mol L^−1^ HNO_3_ and 70% for 1 mol L^−1^ thiourea.

Due to incomplete desorption of Au the slurry sampling graphite furnace atomic absorption spectrometry was proposed for determination of gold in real samples after its enrichment onto **Au(III)/S1**. Detailed information concenrning the desorption studies is presented in the ESM.

### XPS studies

**Fig. 2:**
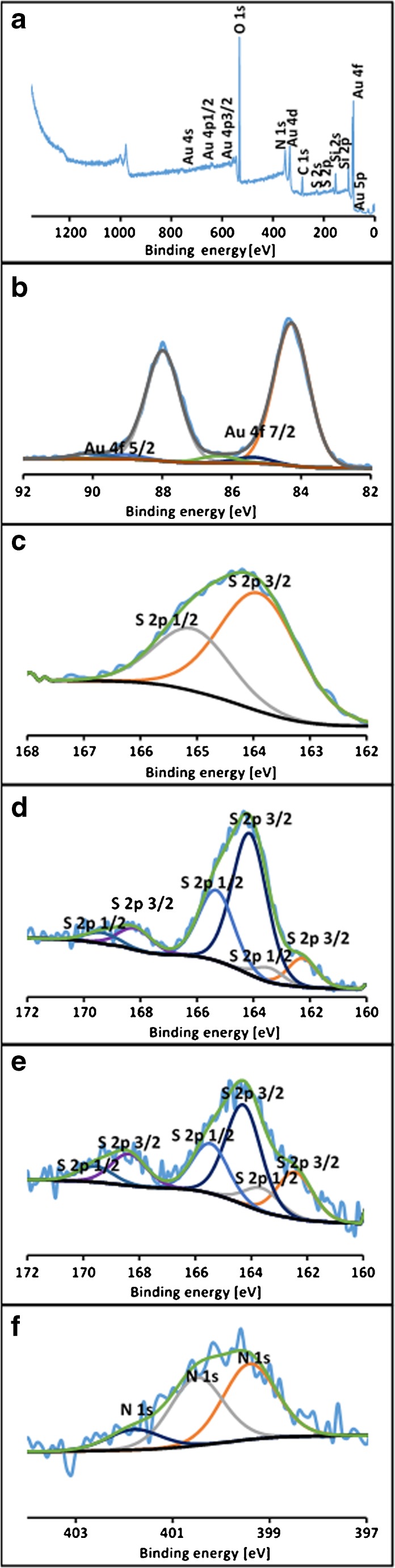
XPS spectra of sample S1, Au(III)S1 and Au-loaded Au(III)S1: XPS spectrum of Au-loaded Au(III)S1 material (**a**); Deconvoluted signals of Au 4f on Au-loaded Au(III)S1 (**b**), S 2p on S1 (**c**), Au(III)S1 (**d**) and Au-loaded Au(III)S1 (**e**), N 1 s on Au-loaded Au(III)/S1(**f**)

In this work XPS was used to investigate the mechanism of the Au(III) ions adsorption onto the thiocyanato-functionalized SBA-15 and their chemical transformations on its surface. In Fig. 2a XPS spectrum for Au-loaded **Au(III)/S1** material is presented. In addition to peaks characteristic for thiocyanato-functionalized silica also peaks assigned to gold are very distinctive. The deconvolution of Au 4f region (Fig. 2b) allows distinguishing the three doublets for Au 4f 7/2 and Au 4f 5/2 transitions at 84.3 and 88.0 eV (first doublet), 85.4 and 89.1 eV (second) and 86.4 and 90.1 eV (third). These correspond to metallic Au, Au(I) and Au(III), respectively. The predominant form was metallic gold, whereas Au(I) and Au(III) was only about 8% of the total Au. Based on the Au 4f region spectrum of Au-loaded **Au(III)/S1**, it can be concluded that the adsorption of Au(III) is associated with its reduction to Au(0). While comparing the S 2p regions of **S1** (Fig. 2c), **Au(III)/S1** (Fig. 2d) and Au-loaded **Au(III)/S1** (Fig. 2e), it can be seen that for **S1** material only sulphur in –C-S-CN groups is present (S 2p 3/2 and S 2p ½ at 163.9 and 165.1 eV, respectively). For two other materials also S bonded with Au (S 2p 3/2 and S 2p ½ at 162.3 and 163.4 eV, respectively) and sulphates (S 2p 3/2 and S 2p ½ at 168.2 and 169.4 eV, respectively) appears wherein the ratio of S^2−^:S^0^:S^6+^ is 12:76:12 for **Au(III)/S1** and 26:56:18 for Au-loaded **Au(III)/S1**. Thus adsorption of Au(III) results in disproportionation of sulphur. Nitrogen irrespective of the type of sorbent is present as amine, imine and quaternary N which is reflected as three peaks in N 1 s region of XPS spectrum. The N 1 s region for Au-loaded **Au(III)/S1** is presented in Fig. 2f.

### Selectivity experiment

To measure the selectivity of the **Au(III)/S1** competitive ion adsorption studies were performed using the double mixture solutions of Au(III)/Pt(IV), Au(III)/Pd(II) and Au(III)/Ru(III). The Pt(IV), Pd(II) and Ru(III) ions were chosen due to their similar ionic properties and ionic radii. The distribution coefficients, selectivity coefficients and relative selectivity coefficients are summarized in Table 2.

**Table 2:** The selectivity parameters of Au(III)TCTES/S1 and TCTES/S1 for Au(III) ions against competitive ions (m = 50 mg, V = 50 mL, CAu(III), Ru(III), Pt(IV), Pd(II) _S1_ = 50 mg L^−1^, CAu(III), Ru(III), Pt(IV), Pd(II) _Au(III)S1_ = 500 mg L^−1^, pH_S1,Au(III)S1_ = 2, t_S1_ = 20 h, t_Au(III)/S1_ = 3 h,*T* = 25 °C)

Ions	Sorbent	K_d_	K_d_(X)	k	k’
		K_d_(Au)			
Au(III)/Pt(IV)	Au(III)/S1	151	0.65	232	72.5
	S1	4.12	1.29	3.20	
Au(III)/Pd(II)	Au(III)/S1	22.0	1.91	11.5	35.8
	S1	3.13	9.73	0.32	
Au(III)/Ru(III)	Au(III)/S1	134	2.15	62.5	16.2
	S1	8.41	2.18	3.85	

The distribution coefficients and selectivity coefficients in ion-imprinted sorbent revealed a significant increase for Au(III) adsorption on Au(III)/S1. Additionally, relative selectivity coefficients were greater than 16, which indicates that **Au(III)/S1** possess very high selectivity towards Au(III) ions.

### Analytical features and application

The calibration plots for gold were obtained using a blank and 5 calibration solutions in the range 5–100 μg L^−1^. The comparison of calibration plot slopes for modified SBA-15 slurries and aqueous solutions confirmed that it was acceptable to use aqueous standards solution for quantitative determination of gold.

The detection limits for gold determination (LOD) (calculated as the average of signals for seven blank samples plus 3 times the standard deviation of the signals) in solution obtained after digestion of samples was 0.01 μg L^−1^ which corresponds to 2 ng g^−1^ in conversion to solid. The characteristic mass (amount providing a signal of 0.0044 s) calculated from the integrated absorbance was 13.8 pg. For a comparison, some previously reported procedures for the determination of gold are summarized in Table 3. As shown, **Au(III)/S1** material is characterized by the highest sorption capacity.

**Table 3:** Comparison of the proposed method with others reported in the literature

Method	Detection technique	LOD[μg L^−1^]	Adsorption capacity [mg g^−1^]	Ref.
Modified magnetic Fe_3_O_4_ –Fir sawdust composite	ICP OES	0.52	188.7	[25]
Ion-exchange polyethylenimine coated on Al_2_O_3_	F AAS	0.0262	6	[11]
Silica gel with rubeanic acid	F AAS	0.80	7.5	[26]
Modified organonanoclay	F AAS	0.1	3.9	[17]
Modified nanostructure inorganic silica	ICP OES	0.11	203.4	[27]
Titanium dioxide nanotubes	ICP MS	0.0013	12.9	[28]
Modified carbon nanotubes	GF AAS	3.1·10^−5^	4.15	[2]
Magnetic nanoparticles	GF AAS	0.16	–	[29]
Fe_3_O_4_@CuS magnetic nanohybrid	F AAS	0.92	333.3	[30]
Ion imprinted polymer coated on multiwalled carbon nanotubes	F AAS	0.041	67	[31]
Imprinted polymer on nanoporous carbon material	F AAS	0.27	81	[32]
Magnetic nanosorbent Fe_3_O_4_/silica/graphene oxide/polypyrrole-polythiophene copolymer	F AAS	0.15	50	[33]
Ion imprinted polymer coated on nanoporous silica	F AAS	0.2	214	[12]
Ion imprinted modified SBA-15	GF AAS	0.01	485.3	This work

The Au(III)TCTES/S1 has been utilized for enrichment and determination of Au in geological samples in view of the great selectivity and sorption capacity for Au(III) ions. The procedure was validated by the application of certified reference materials (Ma-2b, SRM-886, WPR-1, UMT-1 and SARM-7). Additionally, this procedure was applied for determination and preconcentration of gold in magmatic rocks and copper shale samples. The analytical data are shown in Table 4. The precision of gold determination in the geological samples by the presented method can be regarded as acceptable. Moreover, the close agreement of the determined content of Au in reference materials by this method with values reported in the certificates confirms the accuracy of the the method.

**Table 4:** Results of gold determination in CRMs and samples by slurry sampling GFAAS technique after preconcentration onto Au(III)TCTES/S1

Sample	Certified value [mg kg^−1^]	Slurry sampling GF AAS [mg kg^−1^]
Ma-2b	2.39 ± 0.05	2.36 ± 0.12^a^
SRM-886	8.25 ± 0.13	8.85 ± 0.31^a^
WPR-1	0.0422 ± 0.0028	0.0434 ± 0.0096^a^
UMT-1	0.048 ± 0.002	0.051 ± 0.009^a^
SARM-7	0.31 ± 0.015	0.285 ± 0.023^a^
POLK I	–	0.590 ± 0.047^a^
POLK II	–	0.604 ± 0.039^a^
PIG I	–	0.249 ± 0.017^a^
PIG II	–	0.159 ± 0.009^a^
PIG III	–	0.239 ± 0.014^a^
PIG IV	–	0.073 ± 0.008^a^

^a^ standard deviation for five replicate measurements

The method is fairly simple for routine applications and the only limitation found is the time-consuming removal of Au(III) template. However, after a prolonged process of template removal, the obtained material is characterised by high selectivity and extremely high adsorption affinity against Au(III) ions.

## Conclusion

A new selective and sensitive method for determination of trace levels of gold was developed. Au(III)-imprinted thiocyanato-functionalized SBA-15 was used as an adsorbent for gold enrichment from digested geological samples before its determination by slurry sampling GF AAS technique. This new gold ions imprinted material was prepared by a one-pot route synthesis by co-condensation of TEOS with TCTES in the presence of Pluronic123 surfactant and gold ions. The Au(III)/S1 exhibited higher adsorption capacity and selectivity for Au(III) ions in comparison with the control adsorbent prepared in the similar process but without Au(III) ions. The presence of ion-imprinted adsorption sites caused an increase of the rate of Au(III) adsorption and shortened the time needed to reach adsorption equilibrium compared to the control adsorbent. The ion-imprinted material exhibited an extremely high (475 mg g^−1^) adsorption capacity towards Au(III) ions. The adsorption of Au(III) onto ion-imprinted thiocyanato-functionalized SBA-15 was mainly the result of the reduction of Au(III) ions to metallic gold. The application of 10 mol L^−1^ HCl and 14 mol L^−1^ HNO_3_ or thiourea solutions does not cause the complete desorption of gold adsorbed onto studied ion-imprinted organosilica material. Thus slurry sampling GF AAS technique was used for gold determination after its preconcentration onto Au(III)-imprinted thiocyanato-functionalized SBA-15. The method was successfully applied to the analysis of trace gold in geological samples of various matrices. The precision and accuracy of the method were satisfactory for the intended use.

## Electronic supplementary material

ESM 1 (DOCX 288 kb)
