# Supplementary material for: An ion-imprinted thiocyanato-functionalized mesoporous silica for preconcentration of gold(III) prior to its quantitation by slurry sampling graphite furnace AAS
Source: Mikrochim Acta. 2018 Nov 28;185(12):564. doi: 10.1007/s00604-018-3106-x (PMC6267720; doi:10.1007/s00604-018-3106-x)
Supplement: Supplementary file 1 — (DOCX 288 kb) [file 604_2018_3106_MOESM1_ESM.docx]

Electronic Supporting Material on the Microchimica Acta publication entitled

**An ion-imprinted thiocyanato-functionalized mesoporous silica for preconcentration of gold(III) prior to its quantitation by slurry sampling graphite furnace AAS**

**Joanna Dobrzyńska, Marzena Dąbrowska, Rafał Olchowski, Ryszard Dobrowolski**

**Department of Analytical Chemistry and Instrumental Analysis, Faculty of Chemistry, Maria Curie-Sklodowska University, M. C. Sklodowska Sq. 3, 20-031 Lublin, Poland**

**1. Characterization**

## The nitrogen adsorption/desorption isotherms (Fig. S1(A)) of **S1** and **S2** materials have a sharp capillary condensation and evaporation step, which reflect the condensation of nitrogen in uniform mesopores channels and the evacuation of adsorbate from pores. Both isotherms can be classified as type IV, according to the IUPAC classification. The shape of hysteresis loops of the isotherms obtained for Au(III) imprinted materials is completely different and indicates the occurrence of both constricted and open pores. The addition of AuCl_3_ to the reaction mixture results in the collapsed porous structure without predominant mesoporosity.

## Mesostructured organosilicas, albeit with a different degree of order were synthesized under the described conditions. In Fig. S1(B) XRD patterns of studied materials are presented. On the pattern of **S0** (not modified SBA-15) and **S2** samples three peaks are visible in the range of 2θ = 0.8-2°, which can be indexed according to the hexagonal *P6mm* symmetry, indicating the presence of an SBA-15 structure: one reflection at 2θ ≈ 0.9° indexed as (100) and two minor reflections at 2θ≈1.5° and 1.7°, indexed as (110) and (200), respectively. It is notable that the **S2** sample yielded a XRD pattern with lower scattering intensities that indicates relatively poor long-range order in comparison to the **S0** material. In the case of **S1** material only one sharp peak at 2θ ≈ 0.9° was recorded.


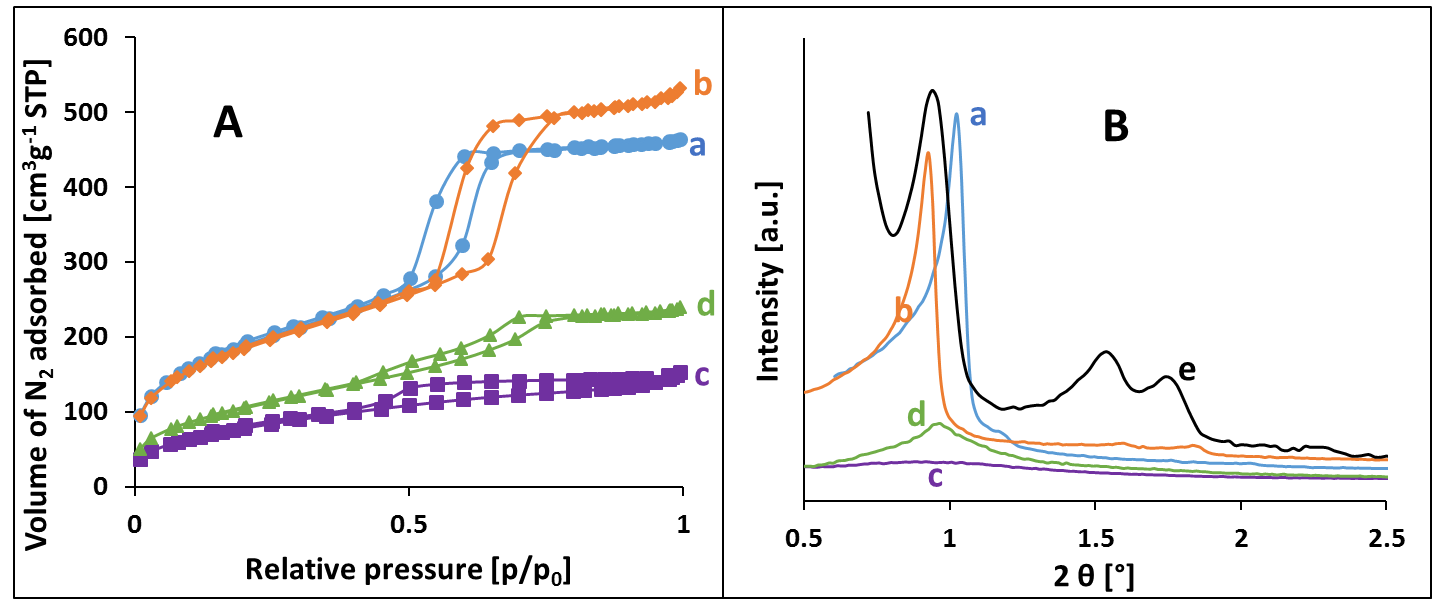


Fig.S1. (A) Nitrogen adsorption/desorption isotherms and (B) XRD patterns of synthesized materials, **a** – S1, **b** - S2, **c –** Au(III)/S1, **d –** Au(III)/S2, **e** – pristine SBA-15.

## **2. Effect of pH**

The pH of the solution is a key factor affecting the adsorption of metal ions because it strongly affects the ionization state of the functional groups of the adsorbent and the chemistry of metal ions. The effect of initial pH on Au(III) adsorption onto the **Au(III)/S1, Au(III)/S2, S1** and **S2** was investigated in the range from 0.5 to 7.0 (which corresponds to the equilibrium pH between 0.5 and 4.2). Fig. S2 shows the relation of ion-imprinted and non-imprinted thiocyanato-functionalized SBA-15 adsorption ability against Au(III) as a function of the equilibrium pH. As evident, adsorption of Au(III) onto **Au(III)/S1** and **Au(III)/S2** is very high across the entire studied pH range. Adsorption of studied ions onto **S1** is also high in the pH range between 2.0 and 4.2, whereas in more acidic conditions it decreases substantially. The adsorption of Au(III) onto **S2** more strongly depends on pH value. A further increase of pH above 3 does not change the adsorption of examined ions onto **S2**. The removal of Au(III) from the solution of pH=0.7 is negligible. The change of pH from 0.7 to 3.0 results in a rapid increase in the adsorption of Au(III) ions onto **S2** material.

In the studied pH range Au(III) exists mainly as AuCl_4_^-^ and AuOHCl_3_^-^ ions, however at pH below 2 also HAuCl_4_ particles without charge coexist in the solution. We suppose that in the case of **S1** and **S2**, thanks to electrostatic forces, only anions are attracted to the positively charged surface and adsorbed. In this case some steric hindrance and lack of attraction between adsorbent surface and HAuCl_4_ molecules may inhibit their adsorption. For ion-imprinted **Au(III)/S1** and **Au(III)/S2** an important factor is the absence of steric hindrance, which facilitates the adsorption of electrostatically neutral molecules from the solutions of low pH.

Fig. S2. The influence of the equilibrium pH on Au(III) adsorption onto: **a** –S1, **b**- S2, **c –** Au(III)/S1, **d –** Au(III)/S2, (m=50 mg, V=50 mL, C_Au_=50 mg L^-1^, t=24 h).

## **3. Effect of the contact time**

Fig. S3 shows the time dependence of the adsorption of Au(III) onto Au(III) imprinted and non-imprinted thiocyanato-functionalized silica materials. The rate of Au(III) adsorption onto **Au(III)/S2** and **S2** is similar, which indicates that ion-imprinting does not improve the overall accessibility of the adsorption centres to the Au(III) anions. For both adsorbents 10 hours are required to reach equilibrium. In the case of the adsorbents synthesized from the TEOS/TCTES mixture in molar ratio 18:2, the adsorption of Au(III) is faster when **Au(III)/S1** material is used. The time needed for reaching equilibrium is about 3 and 20 hours for **Au(III)/S1** and **S1**, respectively. The rapid adsorption onto **S1** in the initial 20 minutes can be attributed to the adsorption on easily accessible adsorption sites. As adsorption progresses its rate decreases, which probably is the result of steric hindrance present in the **S1** material. This hindrance is not observed in **S2** because the concentration of the adsorption sites –SCN is lower than in **S1** and adsorbed ions do not obstruct and repulse each other. The steric hindrance does not affect the adsorption onto **Au(III)/S1** which is attributed to the ion-imprinted adsorption sites shaped as the Au(III) ions, which allows rapid contact of Au(III) ions with bonding atoms.

Fig. S3. Au(III) adsorption kinetics, **a** –S1, **b** - S2, **c –** Au(III)/S1, **d –** Au(III)/S2, (C_Au_=100 mg L^-1^, pH_S1,Au(III)S1,Au(III)S2_=2, pH_S2_=3).

## **4. Effect of interfering ions**

The effect of chlorides and nitrates on the adsorption of Au(III) (Fig. S4) is very important from the analytical point of view due to the common use of *aqua regia* for digestion of analysed solid samples. Taking into account the adsorption capacities, **Au(III)/S1** material was chosen for analytical application. The effect of nitrates in the range between 0.05 and 1 mol L^-1^ is negligible for both **Au(III)/S1** and **S1** adsorbents. The presence of Cl^-^ ions on the level below 0.001 mol L^-1^ causes slight decrease of Au(III) adsorption, for higher (0.1 and 1 mol L^-1^) Cl^-^ concentration adsorption slightly increases. Thus the presence of chlorides and nitrates should not hinder Au(III) adsorption from solutions obtained after acidic digestion of environmental solid samples.


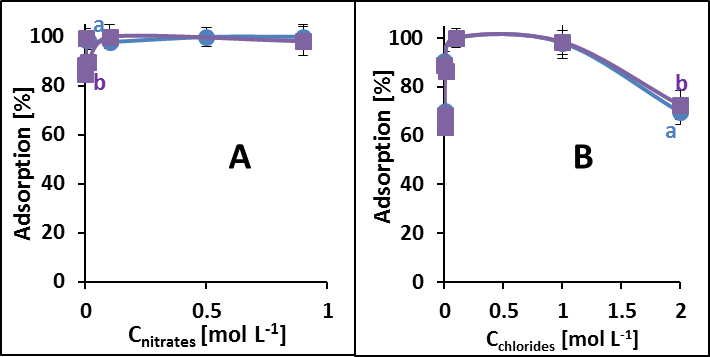


Fig. S4. The influence of (A) NO_3_^-^ and (B) Cl^-^on Au(III) adsorption onto **a** - S1 and **b** - Au(III)/S1; m=50 mg, V=50 mL, t_S1_=20 h, t_Au(III)/S1_=3h, C_Au_=100 mg L^-1^, pH=2.

## **5. Desorption study**

The desorption of Au using different concentrations of hydrochloric acid, nitric acid and thiourea was performed by applying the batch method. As shown in Fig. S5, the efficiency of Au desorption by using proposed inorganic acids is low, with the values obtained for **Au(III)/S1** lower than that for **S1**. The maximum desorption efficiency reached for 10 mol L^-1^ HCl and 14 mol L^-1^ HNO_3_ is about 18 and 33%, respectively. The highest desorption reaching 90% for **S1** and 70% for **Au(III)/S1** materials, was obtained when thiourea was used as the desorptive agent, however total desorption of gold was not achieved. Due to incomplete desorption of Au from examined materials the slurry sampling graphite furnace atomic absorption spectrometry was proposed for determination of gold in real samples after its enrichment onto **Au(III)/S1**.


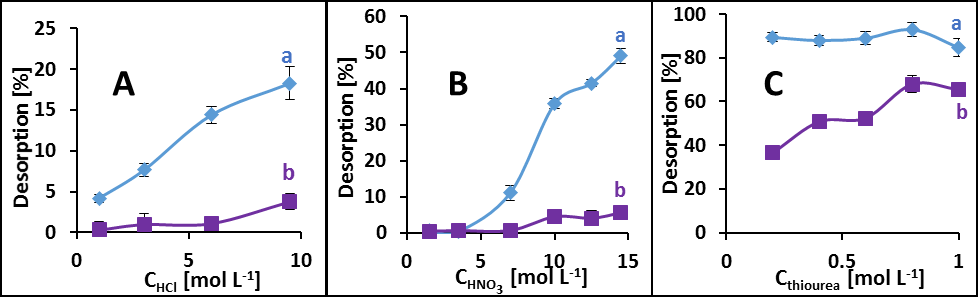


Fig. S5. Desorption of Au(III) from **a** - S1 and **b** - Au(III)/S1; in respect to (A) HCl, (B) HNO_3_ and (C) thiourea concentration (m=1 mg, V=1 mL, t=90 min, T=25°C, A_S1_= 38.7 mg g^-1^, A_Au(III)/S1_= 415.5 mg g^-1^).

**6. Reaction order of Au(III) adsorption**

In order to identify the reaction order of kinetic adsorption of Au(III) onto studied materials all experimental kinetic data were fitted to the usually applied pseudo-first-order equation and pseudo-second-order equation. The Lagergren pseudo-first-order rate equation has the following form:

$ln\left( a_{eq}-a_{t} \right)=lna_{eq}-k_{1}t$ (1)

and the pseudo-second-order equation can be presented as:

$\frac{t}{a_{t}}=\frac{1}{k_{2}a_{eq}^{2}}+\frac{1}{a_{eq}}t$ (2)

where a_t_ (mg g^-1^) is the amount adsorbed at time t. The k_1_ (h^-1^) and k_2_ (g mg^-1^ h^-1^) are the rate constants of the pseudo-first-order equation and pseudo-second-order equation, respectively. The results of the kinetic parameters are shown in Table S1 and presented in Fig. S6. The adsorption of the Au(III) ions is best described by the pseudo-second-order equation for all studied materials, which is confirmed by the correlation coefficient R^2^. It is suggested that the kinetics of metal ions following the pseudo-second-order model is controlled by chemisorption involving sharing or exchanging of electrons between adsorbent and adsorbate.


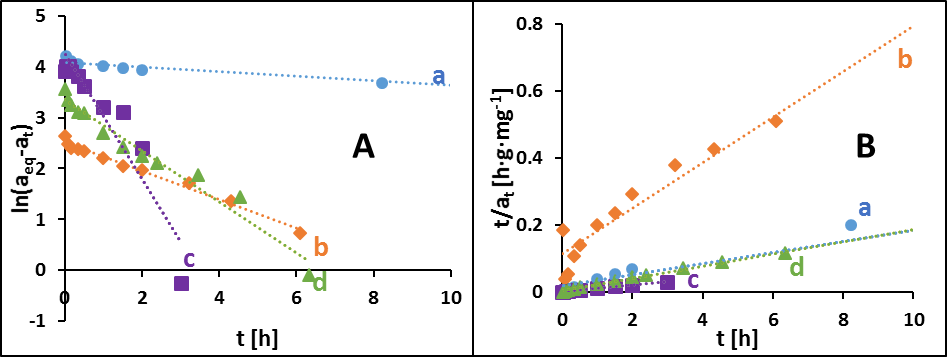


Fig. S6. The kinetic fitting plots according to (A) pseudo-first-order equation and (B) pseudo-second-order equation for Au(III), **a** –S1, **b** - S2, **c –** Au(III)/S1 and **d –** Au(III)/S2.

Table S1. Kinetic parameters for the studied adsorption systems.

| **Materials** | **Pseudo-first-order kinetics** | | | | **Pseudo-second-order kinetic** | | |
| --- | --- | --- | --- | --- | --- | --- | --- |
|  | **a_e_ (exp)**  **[mg g^-1^]** | **k_1_**  **[h^-1^]** | **a_e_ (cal)**  **[mg g^-1^]** | **R^2^** | **k_2_**  **[g mg^-1^ h^-1^]** | **a_e_ (cal) [mg g^-1^]** | **R^2^** |
| S1 | 60.4 | 0.044 | 9.8 | 0.981 | 0.013 | 60.6 | 0.992 |
| S2 | 14.0 | 0.279 | 2.3 | 0.986 | 0.039 | 14.8 | 0.994 |
| Au(III)/S1 | 99.8 | 1.225 | 9.4 | 0.891 | 0.058 | 98.0 | 0.981 |
| Au(III)/S2 | 54.3 | 0.500 | 8.4 | 0.966 | 0.064 | 54.6 | 0.999 |

**7. Langmuir and Freundlich plots for Au(III) adsorption**

Langmuir and Freundlich models were used to fit the adsorption isotherms (Fig. S7). The Langmuir model describes the monolayer adsorption onto homogenous and equivalent adsorbing sites without an interaction between ions adsorbed on adjacent sites. The Langmuir linear equation can be written as follows:

$\frac{C_{eq}}{a_{eq}}=\frac{1}{K_{L}a_{m}}+\frac{C_{eq}}{a_{m}}$ (3)

where C_eq_ is the concentration of adsorbate in equilibrium (mg L^-1^), a_eq_ is the amount of Au(III) ions adsorbed (mg g^-1^), a_m_ is a maximum adsorbed amount required to form a monolayer on the material surface (sorption capacity) (mg g^-1^) and K_L_ represents the Langmuir constant related to adsorption energy (L g^-1^).

The Freundlich model assumes the adsorption onto heterogeneous surface with an interaction between adsorbed ions. The Freundlich isotherm equation is given as follows:

$lna_{eq}=ln\left( a_{m}K \right)+nlnC_{eq}$ (4)

where the Freundlich constant n (0 < n < 1) indicates the intensity of adsorption and characterizes the quasi Gaussian energetic heterogeneity of the adsorption system and K is the Freundlich constant.


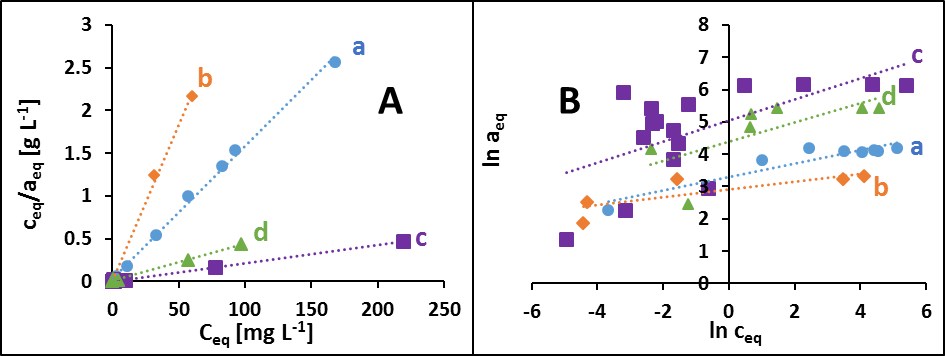
Fig. S7. (A) Langmuir and (B) Freundlich isotherms for the adsorption of Au(III) ions onto **a** –S1, **b** - S2, **c –** Au(III)/S1 and **d –** Au(III)/S2.

Table S2. Parameters of the Langmuir and Freundlich isotherms for Au(III) adsorption.

| **Materials** | **a_exp_ [mg g^-1^]** | **Langmuir** | | | **Freundlich** | | |
| --- | --- | --- | --- | --- | --- | --- | --- |
|  |  | **a_m_ [mg g^-1^]** | **K_L_  [L mg^-1^]** | **R^2^** | **K [(mg g^-1^) (L g^-1^)^n^]** | **n** | **R^2^** |
| S1 | 61.8 | 64.5 | 0.429 | 0.997 | 0.434 | 0.210 | 0.874 |
| S2 | 27.8 | 27.5 | 2.737 | 0.998 | 0.660 | 0.123 | 0.653 |
| Au(III)/S1 | 475 | 476 | 0.75 | 0.997 | 0.324 | 0.327 | 0.384 |
| Au(III)/S2 | 228 | 232 | 0.693 | 0.998 | 0.350 | 0.302 | 0.491 |

Table S2 shows the parameters of the Langmuir and Freundlich isotherms. The Langmuir model gives a better fit than the Freundlich model, which is reflected in higher coefficient factors (R^2^) obtained for the data fitting to the Langmuir equation (Fig. S7). The better correlation with the Langmuir model indicates that Au(III) adsorption onto thiocyanato-functionalized mesoporous silica is a monolayer process. A comparison of the adsorption values calculated from the Eq. 3 with those obtained experimentally suggests that the Au(III) adsorption is equal to the calculated a_m_. Thus the monolayer adsorption is accomplished in all studied materials.
